# Supplementary material for: A Potential Association between Abdominal Obesity and the Efficacy of Humoral Immunity Induced by COVID-19 and by the AZD1222, Convidecia, BNT162b2, Sputnik V, and CoronaVac Vaccines
Source: Vaccines (Basel). 2024 Jan 15;12(1):88. doi: 10.3390/vaccines12010088 (PMC10819553; doi:10.3390/vaccines12010088)
Supplement: Supplementary file 1 [file vaccines-12-00088-s001.zip › vaccines-2755677-supplementary.pdf]

Supplementary material

Table S1. Correlation of BMI with the level of anti-S1/S2 and anti-RBD IgG antibodies.

			BMI/anti-S1/S2 IgG antibodies		BMI/anti-RBD neutralizing antibodies	
**Characteristic**	**Day**	**n**	**Correlation coefficient**	***p*-value**	**Correlation coefficient**	***p*-value**
Prior COVID-19	0	167	0.221	0.004	0.234	0.002
All vaccine	21	338	0.237	<0.0001	0.210	<0.0001
	90	369	0.133	0.010	0.100	0.055
	180	354	0.057	0.284	0.061	0.251
	270	320	0.170	0.0022	-0.056	0.310
	365	302	0.133	0.0199	-0.018	0.745
AZD1222	21	83	0.306	0.005	0.220	0.046
Covidecia	21	74	0.244	0.036	0.253	0.030
Sputnik V	21	72	-	-	0.269	0.022
	90	76	0.272	0.017	0.303	0.008

The data is presented as a correlation coefficient. The p-value refers to a Spearman correlation analysis. The results were considered statistically significant when $p<0.05$.

Table S2. Correlation of age with the level of anti-S1/S2 IgG antibodies and anti-RBD neutralization.

			Age/anti-S1/S2 IgG antibodies		Age/anti-RBD antibodies	
**Characteristic**	**Day**	**n**	**Correlation coefficient**	***p*-value**	**Correlation coefficient**	***p*-value**
Prior COVID-19	0	167	0.252	0.001	0.235	0.002
All	21	338	0.064	0.244	0.136	0.012
	90	369	0.208	<0.0001	0.162	0.002
	180	354	0.084	0.113	-0.064	0.270
	270	320	0.054	0.335	-0.200	0.0003
	365	302	-0.098	0.088	-0.324	<0.0001
Prior COVID-19 AO−	0	54	0.344	0.011	0.309	0.023
Participants AO−	21	148	0.052	0.528	0.166	0.043
	90	159	0.189	0.017	0.161	0.042
	180	151	0.052	0.529	-0.138	0.092
	270	137	0.028	0.742	-0.258	0.0023
	365	116	-0.154	0.098	-0.379	<0.0001
Prior COVID-19 AO+	0	111	0.183	0.055	0.111	0.152
Participants AO+	21	190	-0.140	0.851	0.011	0.880
	90	210	0.155	0.024	0.096	0.164
	180	203	0.069	0.331	-0.017	0.813
	270	183	0.026	0.720	-0.144	0.051
	365	186	-0.086	0.239	-0.275	0.0001
Without prior COVID-19	0	220	0.030	0.658	0.039	0.566
	21	189	0.031	0.675	0.161	0.026
	90	211	0.222	0.001	0.208	0.002
	180	203	0.004	0.959	-0.11	0.119
	270	186	0.038	0.605	-0.180	0.013
	365	165	-0.183	0.018	-0.360	<0.0001
With prior COVID-19	0	167	0.252	0.001	0.235	0.002
	21	149	0.289	<0.0001	0.254	0.001
	90	158	0.370	<0.0001	0.178	0.026
	180	151	0.404	<0.0001	0.102	0.215
	270	134	0.070	0.416	-0.229	0.0076
	365	137	0.020	0.812	-0.266	0.0016

The data is presented as a correlation coefficient. The results were considered statistically significant when $p<0.05$. Abbreviations: AO−, Without Abdominal Obesity; AO+, With Abdominal Obesity.
